# Comparison of the Effects of Intranasal and Oral Midazolam Premedication on Sedation and Separation From Parents for Pediatric Patients

**DOI:** 10.1155/anrp/4837750

**Published:** 2026-05-13

**Authors:** Dilek Metin Yamac, Nedret Erguven, Ecder Ozenc

**Affiliations:** ^1^ Sultan 2. Abdulhamid Han Education and Research Hospital, Department of Anesthesiology and Reanimation, Istanbul, Türkiye; ^2^ Istanbul Haseki Education and Research Hospital, Department of Anesthesiology and Reanimation, Istanbul, Türkiye

## Abstract

**Aim:**

The aim of this study is to compare the effect of intranasal and oral midazolam administered for premedication to children of preschool age on hemodynamic parameters, sedation, and separation from parents.

**Method:**

A prospective, randomized, single‐blind design was adopted, including 60 children aged 2–6 years scheduled for elective surgery under general anesthesia. Participants were randomly assigned into two equal groups using a sealed‐envelope randomization technique (Group O, *n* = 30, 0.5 mg/kg oral midazolam; Group N, *n* = 30, 0.25 mg/kg intranasal midazolam). The study drug was administered 20 min prior to induction of anesthesia. Vital signs and sedation score levels were recorded every 5 min. At the end of the 20th minute, the separation from parents score was assessed and recorded. Assessments were completed by an anesthesiologist who did not know the method of administration for premedication.

**Results:**

There were no statistically significant differences between the two groups in terms of mean age, sex, height, and weight. In Group N, 11/30 (36%) of patients had different types of nasal irritation identified. In both groups, sedation score values examined in the 10th minute were significantly high compared to the initial values. No statistical significance was observed for the other values. There was no statistically significant difference observed for the mean separation from parents scores in Group N and Group O examined in the 20th minute (*p* > 0.05).

**Conclusion:**

In our study, 0.25 mg/kg intranasal midazolam and 0.5 mg/kg oral midazolam, administered to children from 2 to 6 years for premedication, were effective methods, with no significant differences between the two kinds of administration in terms of sedation or separation from parents. Due to the nasal irritation emerging in patients with nasal administration, oral midazolam was evaluated as a more preferable method.

**Trial Registration:** ClinicalTrials.gov: NCT07021755

## 1. Introduction

Careful preoperative assessment and appropriate premedication are essential components of anesthetic management to minimize perioperative morbidity and improve patient outcomes. It has been reported that a substantial proportion of pediatric patients, reaching up to 75%, may experience notable anxiety prior to surgery [1]. Such anxiety may lead to elevated catecholamine levels, resulting in tachycardia, hypertension, and tachypnea, and may also contribute to uncontrolled or aggressive behaviors. Moreover, heightened anxiety has been associated with increased difficulty in managing postoperative pain [2, 3].

Various pharmacological agents are used in pediatric anesthesia practice to alleviate preoperative anxiety and ensure smoother separation from parents and transfer to the operating room [4, 5]. Midazolam, a benzodiazepine derivative, has long been one of the most commonly preferred agents for premedication due to its sedative, anxiolytic, and rapid‐onset effects, as well as its stronger amnestic properties compared with diazepam [2, 3]. Although midazolam can be administered via multiple routes, intramuscular and intravenous administration may be painful and distressing for children [6]. Therefore, selecting a noninvasive, well‐tolerated, and nontraumatic route of administration is essential in pediatric patients, making oral and intranasal routes the most frequently utilized options for preoperative sedation [7].

Although several previous studies have compared different routes of midazolam administration in pediatric patients, most were conducted either using commercially available formulations, atomized intranasal delivery systems, or in settings where alternative sedative agents were readily accessible. Moreover, recent literature increasingly focuses on comparisons between different sedative agents rather than directly examining route‐related differences of the same drug under resource‐limited conditions. The present study provides updated clinical data by directly comparing oral and intranasal administration of the same parenteral midazolam preparation in preschool children, in a real‐world setting where commercially optimized formulations and atomization devices were unavailable. In addition to evaluating sedation and parental separation, this study also addresses tolerability issues such as nasal irritation and their potential methodological implications, thereby contributing practical evidence to guide route selection in similar clinical environments.

The objective of this study was to compare the effects of intranasal and oral midazolam, administered as premedication in preschool‐aged children, on hemodynamic parameters, sedation levels, quality of separation from parents, and the incidence of related complications.

## 2. Methods

This investigation was conducted using a prospective, randomized, single‐blind study design. After receiving permission from the ethics committee of our hospital (Haseki Clinical Research Ethics Committee Centre No: 28.05.2009/10), the study was conducted in accordance with the principles of the Declaration of Helsinki and was registered in a clinical trials registry. A total of 60 pediatric patients aged between 2 and 6 years, classified as American Society of Anesthesiologists (ASA) physical status I‐II and scheduled for elective surgery under general anesthesia, were enrolled. Exclusion criteria comprised known hypersensitivity to midazolam, presence of rhinorrhea, nasal pathology such as polyps, respiratory system disorders, and any neurological or psychiatric conditions. Parents or legal guardians of all participating children were informed in detail about the study protocol, the sedation procedures, potential risks, and benefits. Written informed consent was obtained from the parents or legal guardians of all participants before inclusion in the study. Participation was voluntary, and parents were informed that they could withdraw their child from the study at any time without affecting medical care. The 60 patients included in the study were randomly allocated into two groups using closed envelope method to ensure allocation concealment.

After children were taken to the preoperative assessment section of the operating room accompanied by their parents, they were monitored for electrocardiogram (ECG), noninvasive blood pressure, peripheral oxygen saturation (SpO2), and respiratory count (RC). Basal heart rate (HR), systolic arterial pressure (SAP), diastolic arterial pressure (DAP), mean arterial pressure (MAP), RC, and SpO2 values were recorded before premedication. Twenty minutes before anesthesia induction, the first group was given 0.25 mg/kg intranasal midazolam and named Group N (*n* = 30). Intranasal midazolam was administered by the parent using a needleless 2‐mL syringe, with half the dose given into one nostril and the other half into the other nostril, with the patient in the supine position. The second group was given 0.5 mg/kg oral midazolam and called Group O (*n* = 30). Patients given oral midazolam drank the calculated midazolam dose mixed with 5‐mL pulp‐free fruit juice. Because commercially prepared oral or intranasal midazolam formulations were not available in our institution, parenteral preparation was used for both oral and nasal administration. Intranasal midazolam was delivered using a 2‐mL syringe without an atomizing device due to resource constraints. We acknowledge that this method may have resulted in nonatomized delivery, heterogeneous drug distribution within the nasal cavity, and mild transient discomfort; however, no case of mucosal trauma or bleeding was observed.

After administering the drug, patients had hemodynamic parameters (peak heart rate, SpO2, pressure, and RC) and sedation degree with the Ramsay Sedation Score evaluated every 5 minutes. Sedation scores of 1‐2 were considered inadequate, whereas scores of 3‐4 were regarded as satisfactory. Twenty minutes later, separation from parents was assessed with a four‐stage scale and recorded. For separation from parents, scores of 1 and 2 were satisfactory and scores of 3 and 4 were inadequate. All assessments were completed by an anesthesiologist who did not know which premedication method was applied to the patients.

Ramsay Sedation Scale:

1. Awake; agitated or restless or both
2. Awake; cooperative, oriented, and tranquil
3. Awake but responds to commands only
4. Asleep; brisk response to light glabellar tap or loud auditory stimulus
5. Asleep; sluggish response to light glabellar tap or loud auditory stimulus
6. Asleep; no response to glabellar tap or loud auditory stimulus

Separation from parents scale:

1. Patient unafraid, cooperative, or asleep
2. Slightly afraid/crying, quiet with reassurance
3. Moderately afraid and crying, not quiet with reassurance
4. Crying, need for restraint

Sample size calculation: A priori sample size estimation was performed using the Sealed Envelope sample size calculator, assuming a Type I error (α) of 0.05 and a power of 80% (β = 0.20). Based on an anticipated 30% absolute difference in sedation status at parent separation between the two groups, the minimum required sample size was calculated as 53 patients. An additional margin of approximately 10%–15% was considered to compensate for potential dropouts. Therefore, a total of 60 cases are required for the study (30 in each group).

Statistical analysis: Analysis of data used the SPSS for Windows 10.0 statistical program. Student’s test and Mann–Whitney *U* test were used in the comparisons between the groups; also, “analysis of variance” and “chi‐square test” were used in the repeated measurements.

## 3. Results

Sixty patients were included in the statistical assessment. Demographic characteristics of patients are shown in Table 1. No statistically significant difference was found between the study groups in terms of age, gender, weight, and ASA risk scores (*p* > 0.05) (Table 1).

**TABLE 1:** Age, weight, and sex of cases (mean ± SD).

	**Group N**	**Group O**	**p**

Age (years)	3.97 ± 1.40	3.93 ± 1.39	0.92
Weight (kg)	18.73 ± 15.36	18.73 ± 3.76	0.42
Sex (M/F)	16/14	14/16	0.60
ASA (I/II)	28/2	28/2	1.0

There was no statistically significant difference in terms of mean heart rate in any period between the groups (*p* > 0.05). The heart rate values in Group N fell in the 20th minute compared to values in the 5th, 10th, and 15th minutes (*p* < 0.001). In Group O, the heart rate values in the 20th minute significantly fell compared to the values in the 5th and 10th minutes (*p* < 0.001).

There were no statistically significant differences in mean SAP in any period between the groups (*p* > 0.05). In Group N, the SAP values in the 15th minute were significantly low compared to initial values and values in the 5th and 10th minutes (*p* < 0.001). In Group O, the values in the 20th minute were significantly low compared to values in the 5th, 10th, and 15th minutes (*p* < 0.001). In the 15th minute, values were significantly lower compared to the values in the 5th and 10th minutes. There were no statistically significant differences in terms of mean DAP in any period between the groups (*p* > 0.05). In Group N, the 15th minute values significantly fell compared to the initial values and 5th minute values (*p* < 0.001). In Group N, the 20th minute DAP value was significantly lower compared to the initial values and values in the 5th, 10th, and 15th minutes (*p* < 0.001). In Group O, the 15th minute DAP values were significantly reduced compared to the 5th and 10th minute values (*p* < 0.001). In Group O, the 20th minute DAP values were significantly low compared to the initial values and the values in the 5th, 10th, and 15th minutes (*p* < 0.001).

In Group N, 11/30 patients (36%) were observed to have temporary nasal irritation symptoms like itchy nose, sneezing, and tears. Redness or ulceration was not observed in the postoperative period. In both groups, complications like nausea, vomiting, and nasal irritation were observed and these were treated accordingly (Table 2).

**TABLE 2:** Incidence of complications.

Complication	Group N	Group O	Total
Nausea	1	2	3
Vomiting	1	3	4
Nasal irritation	11	0	11
Hypoxia	0	0	0
Hypertension	0	0	0
Total	13	5	18

There were no statistically significant differences in terms of sedation score values and distribution between the groups in any period (*p* > 0.05) (Table 3). The sedation scores examined in the 20th minute were observed to be at satisfactory levels in Group N and Group O (29/30).

**TABLE 3:** Sedation scale scores.

Sedation scale	Group N (mean ± SD)	Group O (mean ± SD)	*p*
5th min	1.47 ± 0.51	1.47 ± 0.51	1.000
10th min	2.23 ± 0.43	2.07 ± 0.25	0.073
15th min	2.83 ± 0.46	2.73 ± 0.45	0.399
20th min	3.23 ± 0.50	3.20 ± 0.48	0.795

In terms of the separation from parents scale examined in the 20th minute, there were no statistically significant differences observed (*p* > 0.06) (Table 4). While the satisfactory separation scale rate was 90% in Group N in the 20th minute, it was 93% in Group O.

**TABLE 4:** Separation from parents scale scores.

	**Group N (Mean ± SD)**	**Group O (Mean ± SD)**	**p**

Separation from parents scale	1.55 ± (0.63)	1.47 ± (0.63)	0.606

## 4. Discussion

The aim of the study was to investigate the effect of intranasal and oral administration of midazolam for premedication of 2–6‐year‐old pediatric patients on sedation and separation from parents. In previous studies related to the dose of drug used, oral midazolam at 0.5 mg/kg was found to provide more reliable and effective premedication than 0.75 mg/kg and 1 mg/kg doses [7, 8]. Higher doses have not been shown to confer additional clinical benefit and may be associated with an increased risk of adverse effects. As a result, we administered 0.5 mg/kg dose of midazolam orally. For intranasal use, the ideal dose appears to be 0.25 mg/kg, with no benefit found from higher doses [9]. As there is no syrup or spray forms of midazolam in our country, the parenteral form was used in our study. The parenteral midazolam used for oral administration was mixed with 5 mL of pulp‐free fruit juice to improve palatability. Although this volume is small, it may theoretically increase the risk of aspiration in young children with low gastric volumes. No aspiration, regurgitation, or peri‐induction respiratory complications were observed in our study; however, future studies may consider using a smaller volume of liquid or alternative taste‐masking strategies to further minimize this potential risk. For drug administration, Group O was observed to have more comfortable use and easy toleration compared to Group N. In another similar comparative study, the oral midazolam group accepted the drug more easily and was observed to have less anxiety during administration compared to the intranasal midazolam group [7]. Although this study by Deshmukh et al. compared oral and intranasal midazolam in pediatric patients, there are several important differences. In their study, commercially available formulations and more standardized delivery methods were used, whereas in our study, parenteral midazolam was administered via both oral and intranasal routes due to the lack of commercially prepared formulations. Additionally, intranasal administration in our study was performed without an atomization device, reflecting real‐world conditions in resource‐limited settings. Furthermore, our study specifically evaluated tolerability issues such as nasal irritation and discussed their potential impact on blinding and outcome assessment. Therefore, our findings provide complementary and more valuable clinical data that may be particularly relevant in similar healthcare settings.

Nasal irritation was observed with a rate of 36% in our study, whereas it was reported to be observed with a rate of 30% in a study by Bhakta et al. [9]. In another study by Kogan et al. [2], nasal irritation was recorded in 77% of the children. As with other studies, the incidence of nausea and vomiting was low. No group in this study had hypoxia and hypertension observed.

In our study, 36% of children in the intranasal group experienced transient nasal irritation such as sneezing or tearing. The use of a parenteral formulation—rather than a commercially designed intranasal preparation—may have contributed to this effect. Importantly, the occurrence of nasal irritation has methodological implications. Since this symptom is specific to the intranasal route, it may have inadvertently unblinded the assessing anesthesiologist, potentially introducing performance or detection bias in the evaluation of sedation and separation behavior. Although all assessments were conducted by an anesthesiologist who was formally blinded to group allocation, the presence of route‐specific side effects could allow inadvertent inference of treatment allocation. This limitation should be considered when interpreting the subjective outcomes of the study. It is necessary to monitor vital parameters from drug administration until the patient is taken to the operating room [10]. In our study, hemodynamic values (heart rate, SpO2, and HT) were recorded every 5 min for 20 min after sedation, and vital signs were similar in both groups. Another midazolam study investigating oral and intranasal administration observed that vital signs were stable before and after surgery [2, 8].

Previous studies used different sedation scales to determine the sedation score [11, 12]. In our study, we used the 6‐stage Ramsay Sedation Scale. Since the validity of the Ramsay Sedation Score for assessing sedation levels in children was confirmed in the study by Lozana‐Díaz et al. [13], the use of this scale was deemed appropriate in this study. The Ramsay Sedation Scale is a simple tool that is widely used in clinical practice to observationally assess the level of sedation. Limited cognitive and communication skills in preschool children may reduce the reliability of responses to verbal stimuli. In addition, motor responses may be confused with developmental reflexes in this age group, and the level of sedation may be misjudged. This may limit the sensitivity and specificity of the Ramsay Scale in this age group.

Nevertheless, some advantages of the Ramsay Scale may make it preferable in pediatric studies. The fact that it is observation‐based, easy to apply, and widely used in the literature allows comparison with different studies. This scale offers a practical solution, especially in cases where direct communication with children is difficult. Therefore, although the Ramsay Sedation Scale has some limitations in preschool children, its practical use is our reason for preference. However, the preference of age‐specific pediatric scales in future studies will increase the reliability of assessment.

According to the sedation scale, examined every 5 minutes after drug administration, the difference in sedation levels between the two groups was not statistically significant. In the 20th minute, the satisfactory sedation score was 96% in both groups. When similar studies are examined, the 20th minute sedation score was above 75% in both groups [6, 14]. Although a priori sample size calculation was performed based on an expected 30% difference between groups, no statistically significant difference was observed in sedation and separation outcomes. This may indicate that the true effect size between oral and intranasal midazolam is smaller than initially anticipated. Therefore, the study might not have been powered to detect small differences between groups. However, from a clinical perspective, both groups demonstrated high rates of satisfactory sedation (96%) and successful separation from parents (> 90%), suggesting that both routes are similarly effective in routine practice. These findings highlight that the absence of statistical significance does not necessarily imply lack of clinical relevance but rather supports comparable efficacy between the two administration routes.

The most important criterion being a good premedication drug for preschool children is that it makes it easier for the child to separate from his/her parents. In our study, the separation from parents score at the end of the 20th minute in both groups was above 90%. In other studies, the separation from parents score was, respectively, 78% and 89% with 0.5 mg/kg oral midazolam and 91% and 80% with 0.25 mg/kg nasal midazolam [8, 15].

In this study, no statistically significant difference was found between intranasal and oral midazolam administered for premedication in preschool children in terms of sedation level and parental separation behaviors. This finding suggests that both routes of administration may be clinically effective and provide similar levels of patient compliance. Therefore, the premedication route can be chosen flexibly by considering factors such as individual characteristics of children, refusal to take medication, or the experience of the practitioner. However, since the study did not have an equivalence design, it is not possible to say that the two methods have completely equal efficacy. In addition, commenting only on the main clinical outcomes without considering secondary outcomes such as onset time, adverse effects, ease of administration, and patient/parent satisfaction may create a limited perspective. Therefore, an individualized approach in the clinical context gains importance in the selection of these two pathways with similar efficacy.

A comparison with other sedative agents commonly used in pediatric anesthesia, such as dexmedetomidine or nitrous oxide, was not included in this study. The primary aim of the study was to compare two noninvasive administration routes of the same agent, midazolam, which remains one of the most widely used and accessible premedication drugs, particularly in resource‐limited settings. During the study period, alternative sedatives suitable for oral or intranasal administration were not routinely available in our institution, and nitrous oxide sedation could not be applied due to infrastructural limitations. Therefore, the study was intentionally designed to focus on route‐related efficacy, tolerability, and adverse effects of midazolam rather than interdrug comparisons. Future studies may include comparisons with other sedative agents.

### 4.1. Limitations

This study has several methodological and clinical limitations that should be considered when interpreting the findings. First, because commercially prepared oral or intranasal midazolam formulations were unavailable, the parenteral preparation was used for both routes. This may have contributed to the nasal irritation observed in the intranasal group and may limit comparability with studies using atomized or commercially optimized preparations. In addition, intranasal administration using a 2‐mL syringe without an atomizer may have resulted in nonuniform drug distribution and could have influenced patient comfort.

Second, the presence of nasal irritation specific to the intranasal group may have partially unblinded the assessing anesthesiologist despite the single‐blind design, introducing potential detection bias in subjective outcome measures such as sedation and separation scores.

Third, the oral preparation was mixed with 5 mL of juice, which—although clinically uneventful in our population—poses a theoretical risk of aspiration. Future research may explore low‐volume vehicles or commercially available oral formulations.

Finally, our study focused exclusively on the preoperative period. Intraoperative hemodynamic data, onset time of sedative action, postoperative recovery characteristics, and postoperative adverse events (e.g., paradoxical reactions, amnesia, delayed sedation, and respiratory depression) were not assessed. The lack of postoperative follow‐up limits the ability to comprehensively evaluate the safety and efficacy profiles of the two administration routes. However, it is known that recovery and waking are delayed as the midazolam dose is increased, without any difference between the methods in similar studies [7, 16, 17]. Future studies should incorporate postoperative monitoring to provide a more complete clinical assessment.

## 5. Conclusion

This study demonstrated that both intranasal midazolam and oral midazolam are effective and safe premedication methods for preschool children undergoing elective surgery, providing comparable levels of sedation and facilitating satisfactory separation from parents. No significant differences were observed between the two routes with respect to sedation depth, hemodynamic stability, or separation behavior.

However, intranasal administration was associated with a higher incidence of transient nasal irritation, which may negatively affect patient comfort and acceptance. In contrast, oral midazolam was better tolerated and easier to administer, making it a more favorable option in routine clinical practice when oral intake is feasible.

These findings suggest that while both routes can be used successfully, the choice of administration should be individualized, taking into account patient cooperation, tolerance to drug administration, and the clinical setting. Further studies with larger sample sizes, commercially available formulations, and postoperative follow‐up are warranted to better define the optimal premedication strategy in pediatric anesthesia.

## Author Contributions

Concept: Dilek Metin Yamac; literature search: Dilek Metin Yamac; design: Dilek Metin Yamac, Nedret Erguven, and Ecder Ozenc; data collection: Dilek Metin Yamac; analysis and interpretation: Nedret Erguven; manuscript writing: Dilek Metin Yamac and Nedret Erguven; and critical review: Ecder Ozenc.

## Funding

This study was not supported by any fund and/or organization.

## Ethics Statement

This study was approved by the Health Sciences University Haseki Clinical Research Ethics Committee (Approval No: 28.05.2009/10) and conducted in accordance with the Declaration of Helsinki. Written informed consent was obtained from the parents or legal guardians of all participating children prior to inclusion in the study.

## Conflicts of Interest

The authors declare no conflicts of interest.

## Data Availability

The data that support the findings of this study are available on request from the corresponding author. The data are not publicly available due to privacy or ethical restrictions.
